# Population size estimation of seasonal forest-going populations in southern Lao PDR

**DOI:** 10.1038/s41598-021-94413-z

**Published:** 2021-07-20

**Authors:** Francois Rerolle, Jerry O. Jacobson, Paul Wesson, Emily Dantzer, Andrew A. Lover, Bouasy Hongvanthong, Jennifer Smith, John M. Marshall, Hugh J. W. Sturrock, Adam Bennett

**Affiliations:** 1 Malaria Elimination Initiative, The Global Health Group, University of California, San Francisco, CA USA; 2 Department of Epidemiology and Biostatistics, University of California, San Francisco, CA USA; 3 Department of Biostatistics and Epidemiology, School of Public Health and Health Sciences, University of Massachusetts-Amherst, Boston, MA USA; 4 Center for Malariology, Parasitology and Entomology, Ministry of Health, Vientiane, Lao People’s Democratic Republic; 5 Division of Epidemiology and Biostatistics, School of Public Health, University of California, Berkeley, CA USA

**Keywords:** Infectious diseases, Epidemiology, Translational research

## Abstract

Forest-going populations are key to malaria transmission in the Greater Mekong Sub-region (GMS) and are therefore targeted for elimination efforts. Estimating the size of this population is essential for programs to assess, track and achieve their elimination goals. Leveraging data from three cross-sectional household surveys and one survey among forest-goers, the size of this high-risk population in a southern province of Lao PDR between December 2017 and November 2018 was estimated by two methods: population-based household surveys and capture–recapture. During the first month of the dry season, the first month of the rainy season, and the last month of the rainy season, respectively, 16.2% [14.7; 17.7], 9.3% [7.2; 11.3], and 5.3% [4.4; 6.1] of the adult population were estimated to have engaged in forest-going activities. The capture–recapture method estimated a total population size of 18,426 [16,529; 20,669] forest-goers, meaning 61.0% [54.2; 67.9] of the adult population had engaged in forest-going activities over the 12-month study period. This study demonstrates two methods for population size estimation to inform malaria research and programming. The seasonality and turnover within this forest-going population provide unique opportunities and challenges for control programs across the GMS as they work towards malaria elimination.

## Introduction

Malaria transmission in the Greater Mekong Sub-region (GMS) is commonly described as “forest malaria”^1^, and is attributed to the dominance of forest-dwelling malaria vectors such as *Anopheles dirus* and *Anopheles minimus*^2,3^. Activities that result in contact with these vectors in the forest, such as logging, hunting or sleeping, and common forest-fringe activities such as farming or swidden agriculture near forest areas^4–6^ are major risk factors for malaria in the GMS^7–15^ and recent outbreaks in the region have been attributed to deforestation activities^15^. In addition to their increased exposure in the forest, forest-goers are at higher risk for malaria^12^ because of poor adherence to protective measures against mosquitoes and inadequate access to treatment^16^. As malaria declines in the region, most of the residual transmission occurs in forest-going populations that are increasingly targeted for prevention and treatment efforts by national control programs across the GMS^16,17^. Yet, the size of this high-risk population (HRP) remains unknown and difficult to quantify.

Estimating the size of HRPs is important for several reasons^18^. Population size estimates (PSE) can be used to inform policies and mobilize support for control and elimination programs. They are essential to determine the required scale of preventive interventions, to assess intervention coverage, parametrize transmission models, and monitor programs.

There are numerous studies as well as international guidelines^18^ focusing on size estimation of HRPs for HIV^19–24^ but, to our knowledge, none for malaria. In regions where HIV transmission clusters in HRPs, the Second Generation Surveillance (SGS) guidelines for HIV^25^ recommend routine PSE^26^. HRPs for HIV such as sex workers or injecting drug users are considered hard to reach populations because of stigma or discrimination and require sophisticated PSE methods. In malaria, HRPs may not be as hidden, although there may be concerns about the illegal nature of large-scale logging in the GMS^27^. PSE methods, originating in animal ecology, can also be used for non-stigmatized populations and researchers have noted the need for PSE in malaria surveillance where HRPs are key to transmission^28^.

One major difference between forest-going HRPs for malaria in the GMS and most HRPs for HIV is the marked seasonality of their high-risk activities. In this region, the monsoon transforms the environment and affects HRPs’ forest-going activities. For instance, while the rainy season draws populations to rice fields for agriculture, heavy precipitation may also deteriorate roads so that traveling to the forest often becomes challenging. Therefore, evaluating the population size of forest-going HRPs at different time points is essential to identify the appropriate timing of interventions in the GMS.

Identifying HRPs has been key to slow incidence of the HIV epidemic^29–31^ and has relied on surveillance guidelines promoting PSE. In this analysis, we adapted routine HIV surveillance PSE methods to estimate the population size of forest-goers in southern Lao People’s Democratic Republic (PDR), with the goal of improving malaria intervention targeting. Population-based surveys from a randomized controlled trial were used to produce PSEs at three different time points and capture–recapture methodology—drawing on those surveys in addition to a rolling survey of forest-goers—estimated the total number of forest-goers in the study area over the study period.

## Methods

### Study area

**Figure 1:**
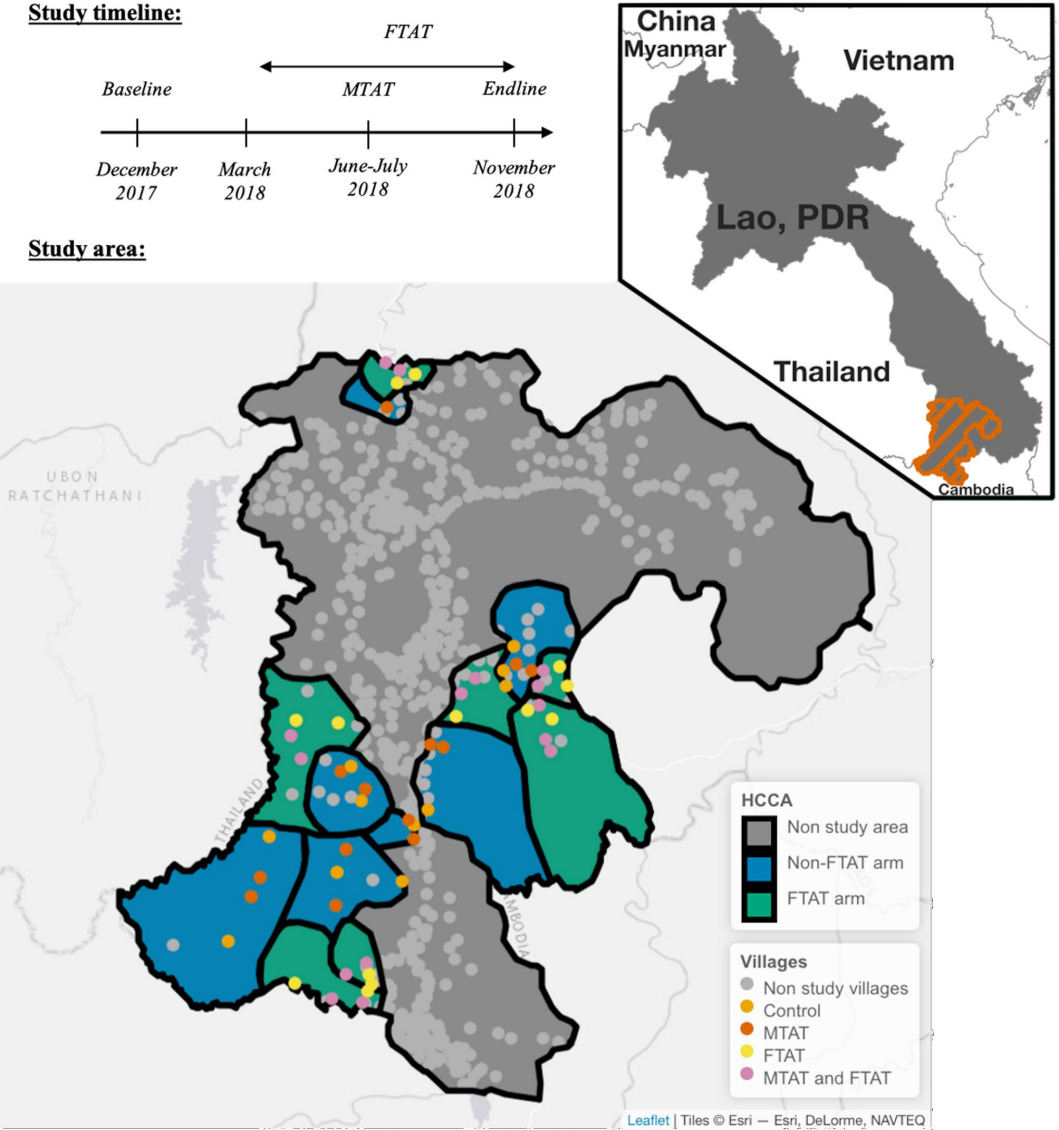
Study timeline and study area: Top left: Study timeline with 3 cross-sectional surveys conducted in December 2017 (Baseline), June–July 2018 (MTAT) and November 2018 (Endline) and a rolling FTAT survey between March and November 2018. Bottom: Study area with 7 of 14 health center catchment areas (HCCA) randomly assigned to FTAT and 28 of 56 villages randomly assigned to MTAT. The study was conducted in Champasak province in southern Lao PDR neighboring Thailand and Cambodia (see upper right indent). The map was produced using leaflet R package with ESRI base map imagery.

This study was conducted in Champasak Province, one of the five southernmost provinces in Lao PDR, together accounting for 95% of the country’s malaria burden^32^. As part of a randomized controlled trial, surveys were conducted between December 2017 and November 2018 to assess the effectiveness of active case detection in village-based and forested-based settings^33^. Across four districts, 56 villages in 14 health center catchment areas (HCCA) were randomized to one of four arms: no intervention, Focal Test-And-Treat (FTAT), an intervention specifically targeting forest-goers, Mass Test-And-Treat (MTAT), where everyone was tested for malaria using rapid diagnostics tests (RDTs) and treated if positive or both interventions. The study area was selected in consultation with the national malaria program based on malaria burden (highest API in 2016). See Fig. 1 for the study timeline and a map of the study area.

The rainy and dry seasons were defined, respectively, as the June to October and November to May periods in consultation with local health ministries and corroborated by actual precipitation data^34^ (see Supplementary Fig. S4.1).

### Population size estimation

We defined the HRP target population as individuals at increased exposure to malaria vectors due to spending the night outdoors for forest or agriculture activities.

In this paper, we report results from two population size estimation methods: population-based household surveys and capture–recapture. The first approach estimated the population proportion of HRP in the study area from three cross-sectional household surveys conducted at different time points during the year. Each proportion was combined with a census count of the total population in the area to produce three distinct PSEs. The capture–recapture methodology drew on individual information from the household surveys and data collected from an intervention among forest-goers conducted over the course of a year to produce another PSE.

These PSEs are complementary but do not estimate the same quantity. The population-based household surveys estimates are “snapshots” of the population size, corresponding to the time frame when the household surveys were conducted. The capture–recapture estimate represents the total population size of HRPs in the study area over the study period, from December 2017 to November 2018. These four estimates would be equal only if, every month, the same HRP individuals spent at least one night outdoors for forest or agriculture activities. If there is seasonality in forest-going, these PSEs should be different.

### Data sources

#### Household census

Over the course of the study, a census of all households and household members in the villages was kept up to date in collaboration with local leaders.

#### Baseline and endline surveys

For the baseline (December 2017) and endline (November 2018) cross-sectional surveys, simple random sampling was used to select 22 and 35 households respectively in each of the 56 study villages. Following written consent, all residents and visitors present in the household at the time of the visit were invited to participate in the survey. Heads of household were asked to answer questions on behalf of absent household members. Primary caretakers answered any questions pertaining to their children when they could not answer themselves. If no householder was at home at time of visit, the study team tried to revisit three times before randomly selecting a replacement household in the village from the household census. The survey was conducted in Lao language by local members of the ministry of health and the national research institution (Lao Tropical Public Health Institute) after receiving comprehensive training^33^. The surveys questioned participants on demographics, forest-going behaviors, treatment-seeking attitudes and malaria knowledge.

#### MTAT survey

Between June 12th and July 23rd 2018, the MTAT intervention was conducted, targeting every household in 28 villages randomly selected from among the 56 villages in the study area. Although questions differed slightly, data collection methods for the survey embedded in this intervention were the same as in the baseline and endline surveys. The study team attempted to visit an absent household three times before marking that household as ‘absent’. The households included in baseline, endline and MTAT were sampled independently from one another^33^.

#### FTAT survey

In the FTAT intervention, conducted continuously between March and November 2018, peer navigators (PNs) were employed in intervention HCCAs to conduct test-and-treat activities amongst members of their communities presumed to be “forest-goers” because of their activities in or near the forest. PNs were themselves forest-goers recruited from the local communities via health authorities and trained to conduct continuous surveillance by testing for malaria using Rapid Diagnostic Tests (RDTs)^33^. PNs were instructed to actively target HRP individuals, and to enroll, once outside the villages, anyone meeting the FTAT HRP eligibility criteria: aged 15 years or older and having spent at least one night outside a formal village in the past 30 days. For 16 HRP individuals interviewed twice in FTAT, we included only data from the first interview.

### HRP eligibility criteria

Participants in the baseline, endline, and MTAT surveys were classified as members of the HRP target population if they were aged 15 years or older, were usual residents of the household, and met any of the criteria listed in Table 1. These criteria were based on responses to survey questions and varied slightly by survey due to differences in questionnaires. All participants in FTAT were classified as HRP due to the intervention’s eligibility criteria; however, we limited the FTAT sample to individuals who reported residing in the study area (56 villages) to ensure geographic alignment with the other surveys.

**Table 1:** HRP eligibility criteria*.*

Baseline and Endline criteria	MTAT criteria
(A) During the past month, stayed overnight away from home AND reason for the absence was working in the rice field, plantation or forest in this province or another province	(D) During the past month, stayed overnight away from home village AND reason for travel was working in a rice field, agricultural or other plantation work, forest foraging, collecting small wood or timber, or logging
(B) Did not sleep in the household the previous night due to working in the rice field, plantation or forest in this province or another province	(E) During the past month, stayed overnight within 10 km of home village AND travel destination was forest, forest fringes, rice field, other field or plantation
(C) Spent at least 1 night in the forest, forest fringe, farms, or rice fields in the past month	(F) Spent at least 1 night in the forest in the past month

### PSE method 1: population-based household surveys

First, we estimated the population proportion of HRP in the study area, *p*_*hrp*_, as the percentage of participants aged 15 years and older in each household survey—baseline, endline and MTAT—that fulfilled the HRP eligibility criteria. Sampling weights and the clustering structure of the respective surveys were specified using the Survey^35^ R^36^ package to correctly estimate population proportions and standard errors.

Second, we developed a pooled estimate of the population proportion of individuals aged 15 and older in households in the study area, *p*_*15*_, by combining, in a meta-analysis using inverse variance, the individual estimates from the 3 surveys.

Third, the total household population in the study area, *Pop,* was obtained by summing the population count listed in the household census across the 56 villages.

Finally, the population-based survey PSE was calculated for each survey as follows:

1 $$PSE={p}_{hrp}\times {p}_{15}\times Pop.$$

The delta method^37^ was used to calculate 95% confidence intervals for each PSE.

The three PSEs obtained from this method pertain to different time periods starting 1 month prior to the first day of the household survey until the last day of the survey (see Table 2).

**Table 2:** Results for the population-based household survey method for population size estimation of HRP individuals.

	PSE time period	Rainy/dry season	% HRP [95% CI]	PSE [95% CI]
Baseline	Oct 28th–Dec 9th 2017	First month of dry season	16.2 [14.7; 17.7]	4898 [4445; 5361]
MTAT	May 12th–July 23rd 2018	First month of rainy season	9.3 [7.2; 11.3]	2801 [2180; 3395]
Endline	Sep 31st–Nov 19th 2018	Last month of rainy season	5.3 [4.4; 6.1]	1586 [1328; 1844]

Two sensitivity analyses were conducted to strengthen the robustness of our results. First, we considered how the differences among criteria may lead to an underestimate of the PSE for the MTAT survey. Second, we attempted to adjust for potential selection bias because of absent households. See Supplementary Materials 5 for details.

### PSE method 2: capture–recapture

Survey participation represented “capture” in the respective survey. To identify participation of the same individual across surveys (i.e., “recapture”), survey records were matched based on age, sex, level of education, first initial and home village. Together, these identifying variables were unique for 99.5% of participants. The matching algorithm allowed plus or minus 2 years for age and 1 level apart for education because rounding age and self-reported education may have introduced errors. See Supplementary Materials 7 for details.

The overlap among the 4 lists of HRP individuals participating in surveys was analyzed using log-linear models^38–42^ by the Rcapture^43^ R^36^ package. The models allowed for temporal dependence due to the potential seasonality of forest-going activities in two ways. First, we estimated a closed population model, where HRP individuals remain in the population all year long but where the probability of being captured differs across surveys because of varying probability of spending a night outside in a given month (M_t_ models). Second, we estimated an open population model, in which HRP individuals may migrate in and out of the population depending on whether or not they spent a night outside in a given month. Both models were designed to estimate the same PSE: the total number of HRP individuals in the study area any time during the 1-year study period from December 2017 to November 2018. See Supplementary Materials 8 for details.

Two sensitivity analyses estimated a lower bound of the PSE by either relaxing the matching criteria or augmenting the eligibility criteria in FTAT. In a third sensitivity analysis, we leveraged the participation of non-HRP individuals in the three household surveys to assess and correct for potential matching errors in the record linkage algorithm. See Supplementary Materials 6 for details.

### Ethics

This study was approved by the National Ethics Committee for Health Research at the Lao Ministry of Health (Approval #2016-014) and by the UCSF ethical review board (Approvals #16-19649 and #17-22577). The informed consent process was consistent with local norms, and all study areas had consultation meeting with, and approvals from, village elders. All participants provided informed written consent; caregivers provided consent for all children under 18, and all children aged 10 and above also provided consent directly. The study was conducted according to the ethical principles of the Declaration of Helsinki of October 2002.

## Results

### Data description

#### Household-based surveys

In the baseline, MTAT and endline surveys respectively, 5723, 18,143 and 7870 individuals across 1310, 4489 and 2081 households, were interviewed. Responses required to construct HRP criteria were provided by 99.6%, 97.4%, and 99.9% of baseline, MTAT and endline participants, respectively (see Supplementary Tables S1.1–3).

Of those 47,575 inhabitants living in the study area—*Pop* in Eq. (1), 63.5% (95% CI [62.9%; 64.2%]) were estimated to be older than 15 years—*p*_*15*_ in Eq. (1). See Supplementary Materials 3 for details.

#### FTAT survey

Among the 2888 HRP individuals recruited into the FTAT survey, 2305 (79.8%) came from one of the 56 villages in our study area and were included in this study. Supplementary Fig. S4.2 shows the weekly enrollment.

**Figure 2:**
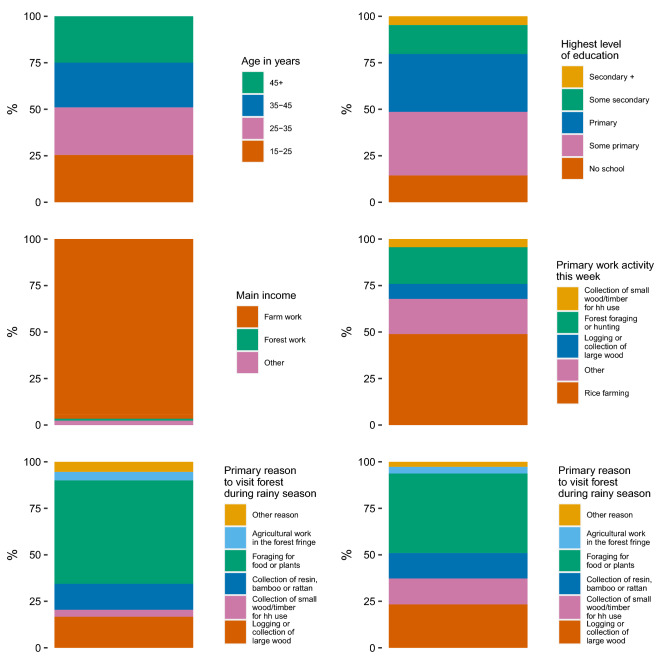
Demographics of FTAT HRP: age, education, income, work activity, reasons to visit the forest of HRP individuals enrolled in FTAT survey.

Figure 2 shows the distribution of selected variables from the FTAT survey. Males were more represented (67.2%) than females (32.8%) and the average age was 36.4 years. A majority (96.6%) of HRP individuals earned their primary income from agricultural work and about 50% reported rice farming as their primary activity. The proportion of HRP individuals reporting the collection of wood as the primary reason to visit the forest almost doubled between the rainy (20.4%) and dry (37.3%) seasons.

**Figure 3:**
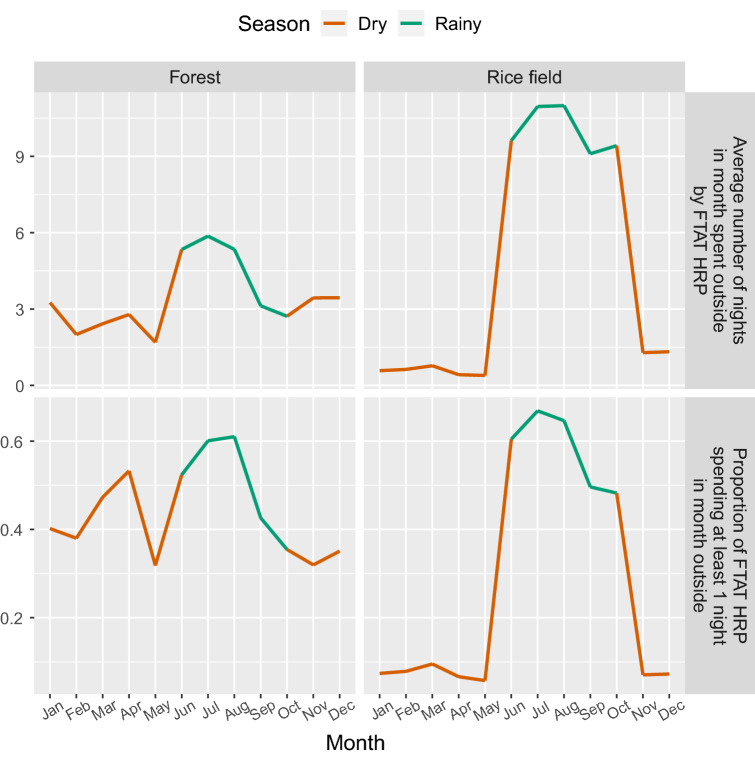
Seasonality of FTAT HRP: Top row—average number of nights spent outside in the forest or rice field by FTAT HRP individuals over time. Bottom row—proportion of FTAT HRP individuals spending at least 1 night in month outside in the forest or rice field over time.

**Figure 4:**
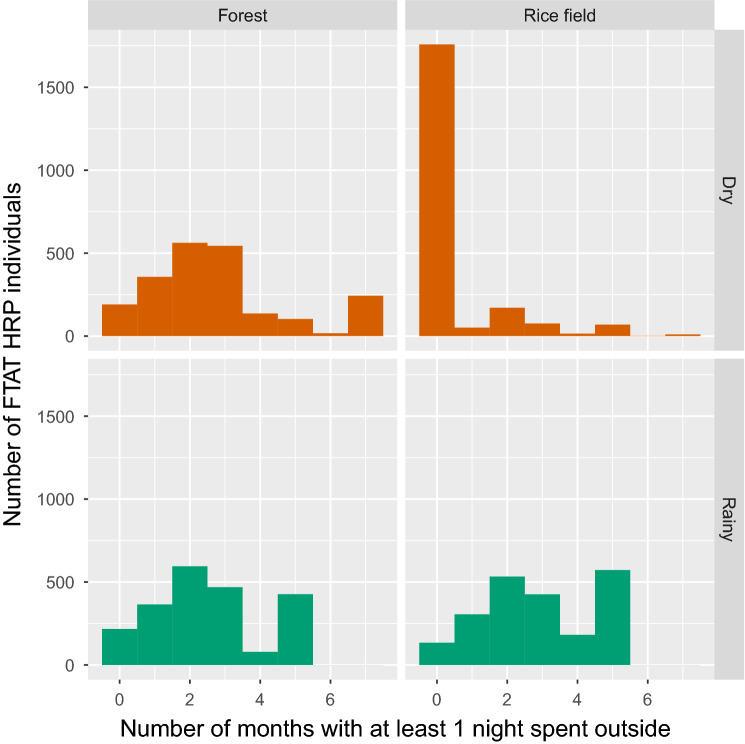
Turnover of FTAT HRP: distribution of the number of months in which FTAT HRP individuals reported spending at least 1 night outside in the forest or rice field during the rainy (5 months between June and October) and dry (7 months between November and May) season.

The number of nights typically spent outside each month of the year was reported by FTAT HRP individuals and summarized in Figs. 3 and 4. During the rainy season, activities in the rice field intensified, with about 60% of HRP individuals spending at least one night outside in any given month and about 10 nights per month spent outside on average. During the dry season, few HRP individuals reported spending a night in the rice field. In contrast, forest-going was characterized by a greater average number of nights and a greater proportion of HRP individuals spending a night outside during the rainy season and occurred more regularly throughout the year. Across all months, at least 30% of HRP individuals reported spending at least 1 night in the forest. These plots also suggest a high level of turnover with many HRP individuals reporting spending nights outside in only 1–3 months of either the dry or rainy season.

### PSE method 1: population-based household surveys

Table 2 presents the estimated population proportion of HRP and the resulting PSE from each of the three cross-sectional household surveys.

### PSE method 2: capture–recapture

**Figure 5:**
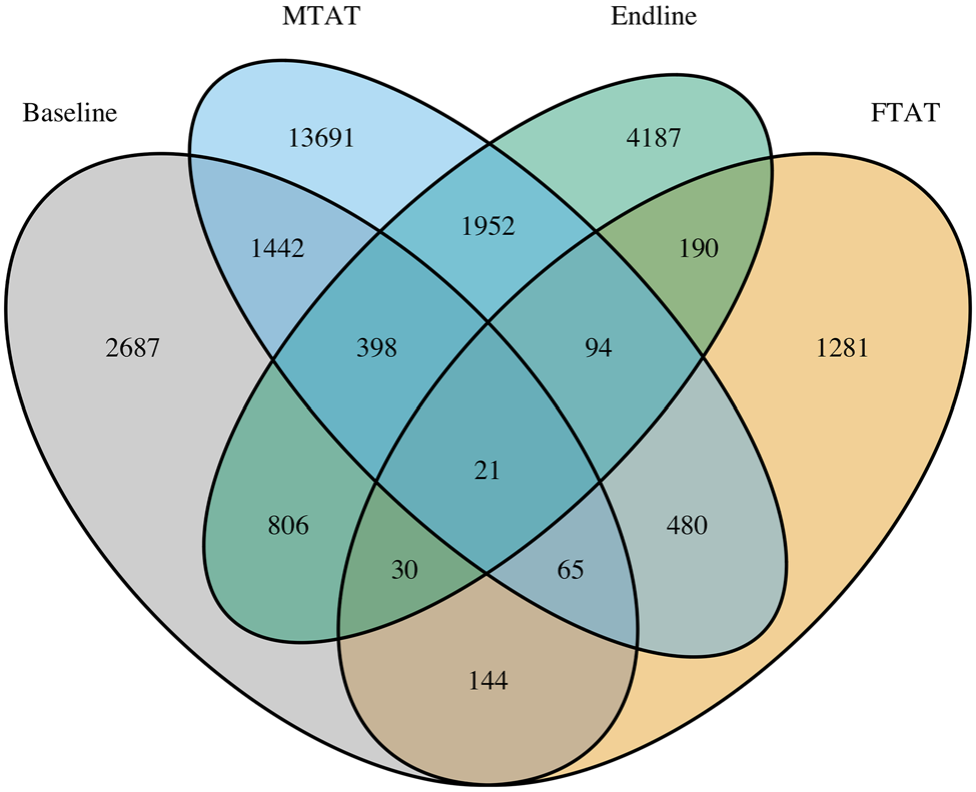
Capture history: Venn Diagram of the capture history data. For instance, 128 HRP individuals were captured both in the MTAT and FTAT surveys but not in baseline or endline surveys.

A total of 557, 1040, 269 and 2305 HRP individuals from the study area were captured in the baseline, MTAT, endline and FTAT surveys, respectively. After matching participants, 3869 unique HRP individuals were identified across the four surveys. Figure 5 presents a Venn Diagram of these capture history data.

Table 3 shows the capture–recapture results from log-linear models fit to our data, assuming a closed population. Models that allowed for correlation of the probability of selection across surveys (denoted M_b_), did not perform well. Their fit to the data, as indicated by the AIC and BIC, was poor and their PSEs were barely above 3869, the total number of unique HRP individuals captured. The model allowing for temporal dependence (M_t_) yielded the best fit in terms of both AIC and BIC. Allowing for heterogeneity (M_h_) between individuals in terms of their selection probability did not result in a better fit in any parametrization (i.e., Chao, Poisson2, Darroch or Gamma 3.5). Additionally, Supplementary Materials 9 details a diagnostic test that determined it was not necessary to incorporate heterogeneity in the models.

**Table 3:** Capture–recapture PSE results using log-linear models and assuming closed population.

	PSE	Standard error	Deviance	Df	AIC	BIC
M0	20,892.7	1103.9	2384.2	13	2461.4	2474
Mt	17,106.6	878.1	23	10	106.3	137.6
Mh Chao (LB)	21,136.3	1192.3	2383.8	12	2463.1	2481.9
Mh Poisson2	22,317.5	3240.9	2383.9	12	2463.2	2482
Mh Darroch	24,222.1	6664.4	2383.9	12	2463.1	2481.9
Mh Gamma3.5	26,272.7	10,795.7	2383.8	12	2463.1	2481.9
Mth Chao (LB)	17,476	953.1	21.4	9	106.7	144.3
Mth Poisson2	19,900	2782.9	21.7	9	107	144.6
Mth Darroch	23,693.6	6493.3	21.6	9	106.9	144.4
Mth Gamma3.5	28,267.4	11,782.7	21.5	9	106.8	144.4
Mb	6052.6	210.2	2182.4	12	2261.7	2280.5
Mbh	3998.6	30.7	200.2	11	281.5	306.6

Profile likelihood^44^ was used to calculate a 95% CI of [15,502; 18,959] for the M_t_ capture–recapture PSE of 17,107. Changing our conceptual framework for temporal dependence and modeling an open population did not improve the fit to our data (AIC = 108.2) and yielded a similar PSE of 17,008 [15,136; 18,880]. In our final M_t_ model, an additional interaction term between baseline and MTAT improved the fit (AIC = 93.0) and led to a final PSE of 18,426 [16,529; 20,669], representing 61.0% [54.2; 67.9] of the household population aged 15 years or older in the study area (using Eq. 1). See Supplementary Materials 2 for additional results.

## Discussion

Based on data from a randomized controlled trial conducted between December 2017 and November 2018, we applied two methods to estimate the number of forest-goers in Champasak province in southern Lao PDR. Leveraging the different timing of three cross-sectional household surveys, forest-going HRPs were found to represent 16.2% [14.7; 17.7], 9.3% [7.2; 11.3], and 5.3% [4.4; 6.1] of the household population older than 15 years during the first month of the dry season, the first month of the rainy season, and the last month of the rainy season, respectively. The capture–recapture method estimated a total population size of 18,426 [16,529; 20,669] forest-goers present at any time over the period, representing 61.0% [54.2; 67.9] of the population 15 years or older.

A key finding from this study is that a large majority of adult residents in the study area spent at least one night outdoors for forest or agricultural activities over the course of a year. This has important implications for malaria control programs, suggesting they may have underestimated the size of forest-going populations that are increasingly targeted by prevention and treatment efforts^16,17^. In particular, focal forest-based interventions may help better target forest-goers at higher-risk for malaria than village-based self-reported forest-goers and programs should scale-up the delivery of forest-goers’ packages with spatial repellents or insecticide treated portable hammocks. Alternatively, these results call for a more stringent definition of the forest-going label to identify higher-risk forest-goers as there may be variability in the associations between HRP eligibility criteria in Table 1 and malaria. As highlighted in a recent systematic review of the qualitative literature on forest-goers in the GMS^45^, a better characterization of the activities that put forest-goers at increased risk for malaria is needed. This is critical to clearly identify and count HRPs.

Data from the FTAT survey showed that forest-going HRP individuals were much more active during the rainy season, especially in the rice fields. In contrast, the household surveys identified a greater number of forest-goers during the dry season than the rainy season. Yet, this difference may be an artifact of selection bias since twice as many households approached during the rainy season (i.e. in the MTAT survey) could not be enrolled due to householders being away, compared to the dry season (i.e. in the baseline survey). Anecdotal evidence from field teams suggests that households were often vacant because household members were working in the forest or at agriculture sites. That said, sensitivity analyses found that no more than 25% of the population had spent a night outside for forest or agricultural activities in a given month during either the rainy or dry season. This implies a high turnover among the forest-going HRPs with individuals spending a night outside for forest or agriculture activities only in certain months of the year. Seasonality and turnover thus appear to be important considerations when designing interventions to access and treat these forest-going HRPs. For instance, our results show a drop in the number of forest-goers active toward the end of the rainy season which could be leveraged by interventions to more effectively target forest-goers both in the forest and in the villages, where many may have already returned.

In our statistical models, the closed population assumption was most consistent with the data, suggesting there was no change in the HRP population over the 1-year study period. The seasonality and turnover among forest-going HRPs highlighted in the FTAT data and, additionally, by variation across the household-based PSEs, was accommodated in closed population models M_t_ with a capture probability allowed to vary among surveys. Another way to account for this temporal dependence was to change our conceptual framework and restrict our HRP definition to individuals spending a night outside for forest or agriculture activities in a *given month* of the 1-year study period. As the number of HRP individuals can now vary between 2 months, open population models were used. Importantly though, we considered this alternative approach to estimate the same PSE, i.e. the total population size of HRPs in the study area during the study period. Results from both conceptual frameworks were consistent.

Routinely used in HIV surveillance^25^, PSEs could strengthen the malaria surveillance arsenal^28^. Population-based surveys, unsuitable to identify hidden and hard-to-reach HRPs in HIV, are simple methods frequently used in malaria research that could be leveraged for PSEs of forest-goers in the GMS and other high-risk subgroups such as cattle-herders in southern Africa^46^. As illustrated here, in the presence of seasonal risk behaviors, PSEs reflecting different periods over the malaria season can be obtained by conducting multiple surveys at different time points. A cumulative PSE can be obtained by applying capture–recapture to three or more data sources, ideally including a longitudinal survey, such as our FTAT survey. Additional sources might include surveillance data routinely collected by malaria programs or more targeted data such as surveys at known venues where HRP congregate. Equipped with routine PSEs, malaria control program could better serve their respective HRP. In the GMS for instance, the spatio-temporal variability in forest-going populations size estimates can inform where and when to target specific quantities for interventions such as insecticide treated hammock nets or chemoprophylaxis.

Our results do have limitations. First, the study did not use a standardized definition for forest-going HRPs across surveys. To address this issue, we combined multiple survey questions to identify HRP individuals. Our sensitivity analysis estimated a possible 17% undercount in the MTAT survey. Second, our HRP definition lumps together night spent outdoors for forest and agriculture activities. In Supplementary Information 10, we attempted to distinguish forest from agriculture activities, but the wording of surveys questions did not allow for a clear distinction and resulted in ranges of estimates too wide to be informative. Third, asking heads of households to answer question items to assess HRP criteria on behalf of absent household members may have contributed to more complete data, but also to misclassification. Our formative work indicated forest and agricultural activities were not significantly stigmatized in the study area so this should not have led to a meaningful bias. Lastly, individuals could not be matched across surveys on full names so that initials were used, potentially leading to matching errors. In a sensitivity analysis, captures of non-HRP individuals in household surveys were leveraged to evaluate that such matching errors could have led to no more than an 11% undercount in our capture–recapture PSE.

In conclusion, this study estimated the overall proportion that forest-going HRPs represent in southern Lao PDR, highlighted an important seasonality in malaria risk behaviors, and illustrates population size estimation methods that can be replicated to support national malaria programs in the GMS to assess and meet their malaria elimination goals^47,48^.

## Supplementary Information

Supplementary Information 1.

Supplementary Information 2.

Supplementary Information 3.

## Data Availability

The datasets generated and analyzed during the current study are publicly available and shared along the article submission (Supplementary Information 2 and 3). Personal identifying variables were removed to protect anonymity of study participants. The capture recapture codes rely on these variables and therefore won’t work but are made publicly available for transparency. Feel free to reach out to the corresponding authors for additional information.
