# Publisher Correction: Population size estimation of seasonal forest-going populations in southern Lao PDR

**DOI:** 10.1038/s41598-023-30416-2

**Published:** 2023-02-27

**Authors:** Francois Rerolle, Jerry O. Jacobson, Paul Wesson, Emily Dantzer, Andrew A. Lover, Bouasy Hongvanthong, Jennifer Smith, John M. Marshall, Hugh J. W. Sturrock, Adam Bennett

**Affiliations:** 1 Malaria Elimination Initiative, The Global Health Group, University of California, San Francisco, CA USA; 2 Department of Epidemiology and Biostatistics, University of California, San Francisco, CA USA; 3 Department of Biostatistics and Epidemiology, School of Public Health and Health Sciences, University of Massachusetts-Amherst, Boston, MA USA; 4 Center for Malariology, Parasitology and Entomology, Ministry of Health, Vientiane, Lao People’s Democratic Republic; 5 Division of Epidemiology and Biostatistics, School of Public Health, University of California, Berkeley, CA USA

Correction to: *Scientific Reports*, 10.1038/s41598-021-94413-z, published online 20 July 2021

In the original version of this Article, the Supplementary Information 2 and 3 were omitted.

The Supplementary Information files now accompany the original Article.

As a result, the Data availability section was revised.

“The datasets generated and analyzed during the current study are publicly available and shared along the article submission. First name initials of study participants were coded numerically to anonymize them.”

now reads:

“The datasets generated and analyzed during the current study are publicly available and shared along the article submission (Supplementary Information 2 and 3). Personal identifying variables were removed to protect anonymity of study participants. The capture recapture codes rely on these variables and therefore won’t work but are made publicly available for transparency. Feel free to reach out to the corresponding authors for additional information.”

The original Article has been corrected.

