# Supplementary material for: Population size estimation of seasonal forest-going populations in southern Lao PDR
Source: Sci Rep. 2021 Jul 20;11:14816. doi: 10.1038/s41598-021-94413-z (PMC8292394; doi:10.1038/s41598-021-94413-z)
Supplement: Supplementary file 1 — Supplementary Information 1. [file 41598_2021_94413_MOESM1_ESM.pdf]

Supplementary Materials 1: additional tables for population-based household survey method

Measure	N	n	% [95% CI]	Missing observations n (%)
Usually resident in HH	5,723	5,593	97.3 [96.5; 98.0]	1 (0)
Age older than 15 among usual residents	5,593	3,378	60.2 [59.1; 61.3]	2 (0)
HRP criteria:				
A	3,378	383	11 [9.7; 12.4]	1 (0)
B	3,378	138	3.9 [3.0; 4.7]	5 (0.1)
C	3,378	443	13.1 [11.7; 14.4]	8 (0.2)
Any of A, B, C	3,378	557	16.2 [14.7; 17.7]	13 (0.4)

Table S1.1 - *Identification of HRP individuals in baseline survey*

Measure	N	n	% [95% CI]	Missing observations n (%)
Age older than 15	18,143	11,526	63.5 [62.0; 65.1]	5 (0)
HRP criteria:				
D	11,526	879	7.8 [6.0; 9.7]	280 (2.4)
E	11,526	831	7.4 [5.6; 9.2]	277 (2.4)
F	11,526	284	2.5 [1.5; 3.6]	288 (2.5)
Any of D, E, F	11,526	1,040	9.3 [7.2; 11.3]	302 (2.6)

Table S1.2 - *Identification of HRP individuals in MTAT survey*

Measure	N	n	% [95% CI]	Missing observations n (%)
Usually resident in HH	7,870	7,678	97.5 [97.0; 98.1]	1 (0)
Age older than 15 among usual residents	7,678	5,023	65.9 [64.9; 66.8]	0 (0)
HRP criteria:				
A	5,023	189	3.7 [3.0; 4.5]	0 (0)
B	5,023	85	1.6 [1.1; 2.0]	0 (0)
C	5,023	215	4.3 [3.5; 5.0]	1 (0)
Any of A, B, C	5,023	269	5.3 [4.4; 6.1]	1 (0)

Table S1.3 - *Identification of HRP individuals in endline survey*

Supplementary Materials 2: additional tables for capture-recapture method

Number of lists	Surveys	PSE [95% CI]
4	FTAT, MTAT, baseline, endline	17,107 [15,502; 18,959]
3	FTAT, MTAT, baseline	16,999 [15,241; 19,061]
	FTAT, MTAT, endline	17,247 [15,221; 19,679]
	FTAT, baseline, endline	19,578 [16,459; 23,532]
	MTAT, baseline, endline	12,946 [10,594; 16,089]
2	FTAT, MTAT	17,371 [15,012; 20,295]
	FTAT, baseline	21,047 [16,836; 26,886]
	FTAT, endline	16,317 [12,387; 22,256]
	MTAT, baseline	10,344 [8,216; 13,333]
	MTAT, endline	19,983 [12,567; 34,880]
	baseline, endline	21,405 [11,238; 49,131]

Table S2.1 - *Capture-recapture M_t PSE using 2, 3 or 4 of the survey lists available*

Model	PSE [95% CI]	AIC
Closed population M_0	20,886 [18,877; 23,223]	2461.44
Closed population M_t	17,107 [15,502; 18,959]	106.30
Open population	17,008 [15,136; 18,880]	108.23
**Final model: Closed population M_t with baseline-MTAT interaction**	**18,426 [16,529; 20,669]**	**92.97**

Table S2.2 - *Capture-recapture PSE for various models considered*

	PSE	Standard Error	Deviance	Df	AIC	BIC
Baseline & MTAT	18,425.9	1,050.7	7.7	9	93.0	130.5
Baseline & MTAT; Baseline & FTAT	17,640.5	1,139.2	5.9	8	93.2	137.0
Baseline & MTAT; FTAT & MTAT	19,703.4	1,681.1	6.5	8	93.8	137.6
No interaction	17106.6	878.1	23	10	106.3	137.6
Baseline & MTAT; FTAT & Endline	18,788.3	1,160.9	7.0	8	94.2	138.1
Baseline & MTAT; Baseline & Endline	18,361.6	1,058.3	7.5	8	94.8	138.6
Baseline & MTAT; MTAT & Endline	18,354.3	1,071.8	7.6	8	94.9	138.7
Baseline & FTAT	16,173.5	914.4	18.7	9	104.0	141.6

Table S2.3 - *Capture-recapture PSE for M_t models with additional interaction terms between surveys*

Supplementary Materials 3: additional table for meta-analysis estimating p_{15}

	Survey period	Age older than 15 (%) [95% CI]	Age older than 15 (SE)
Baseline	Nov 28th – Dec 9th 2017	60.2 [59.1; 61.3]	0.00577
MTAT	June 12th – July 23rd 2018	63.5 [62.0; 65.1]	0.00794
Endline	Oct 31st – Nov 19th 2018	65.9 [64.9; 66.8]	0.00485
Overall		63.5 [62.9; 64.2]	0.00336

Table S3.1 - *Meta-analysis to estimate proportion of population older than 15*

Supplementary Materials 4: Additional figures

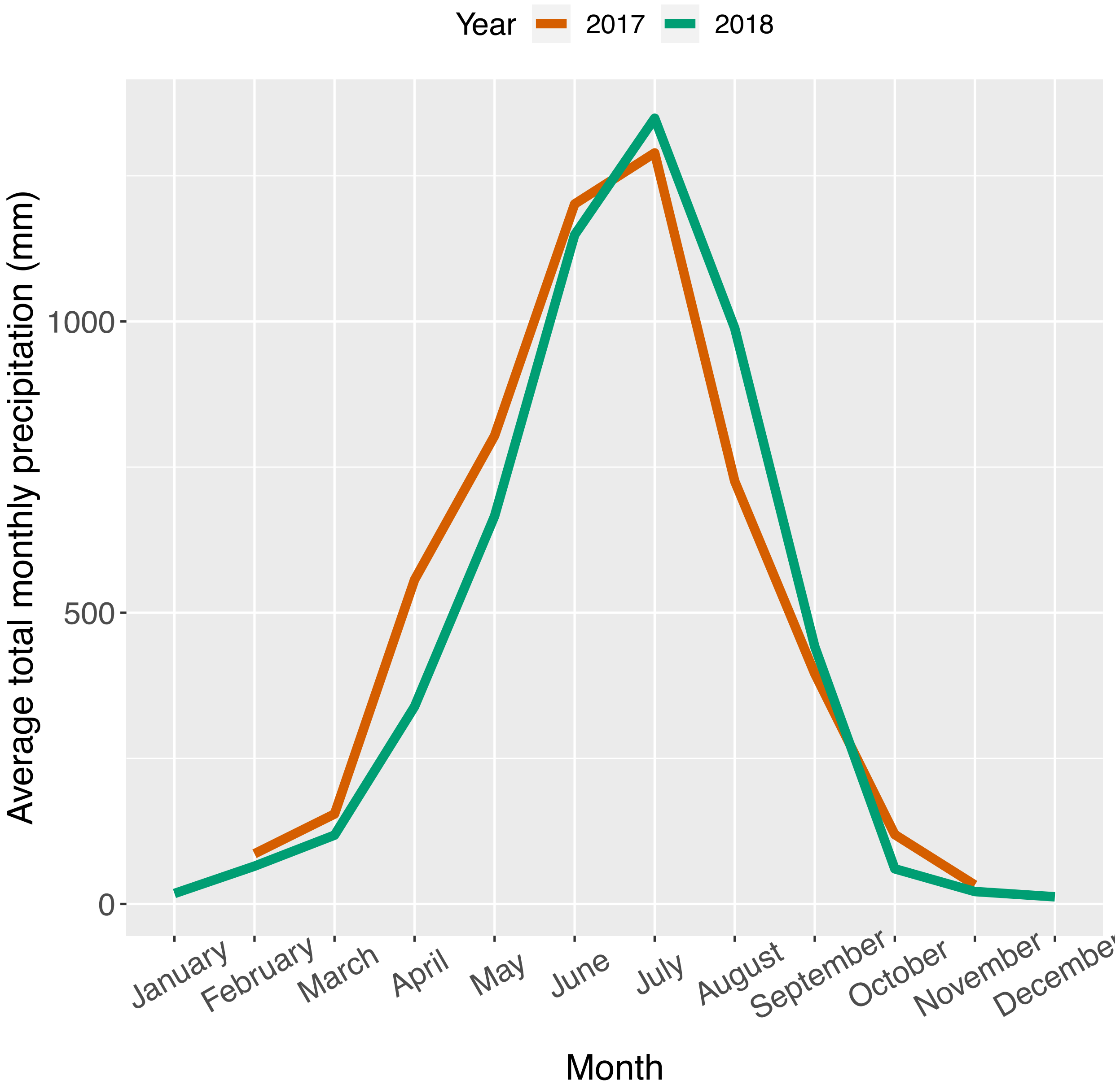

Figure S4.1 – Preciptiation time series: *Average total monthly precipitation (mm) in Champasak, southern Lao PDR. Precipitation data from CHIRPS in all 300m2 pixels of Champasak province were averaged for all months of 2017 and 2018.*

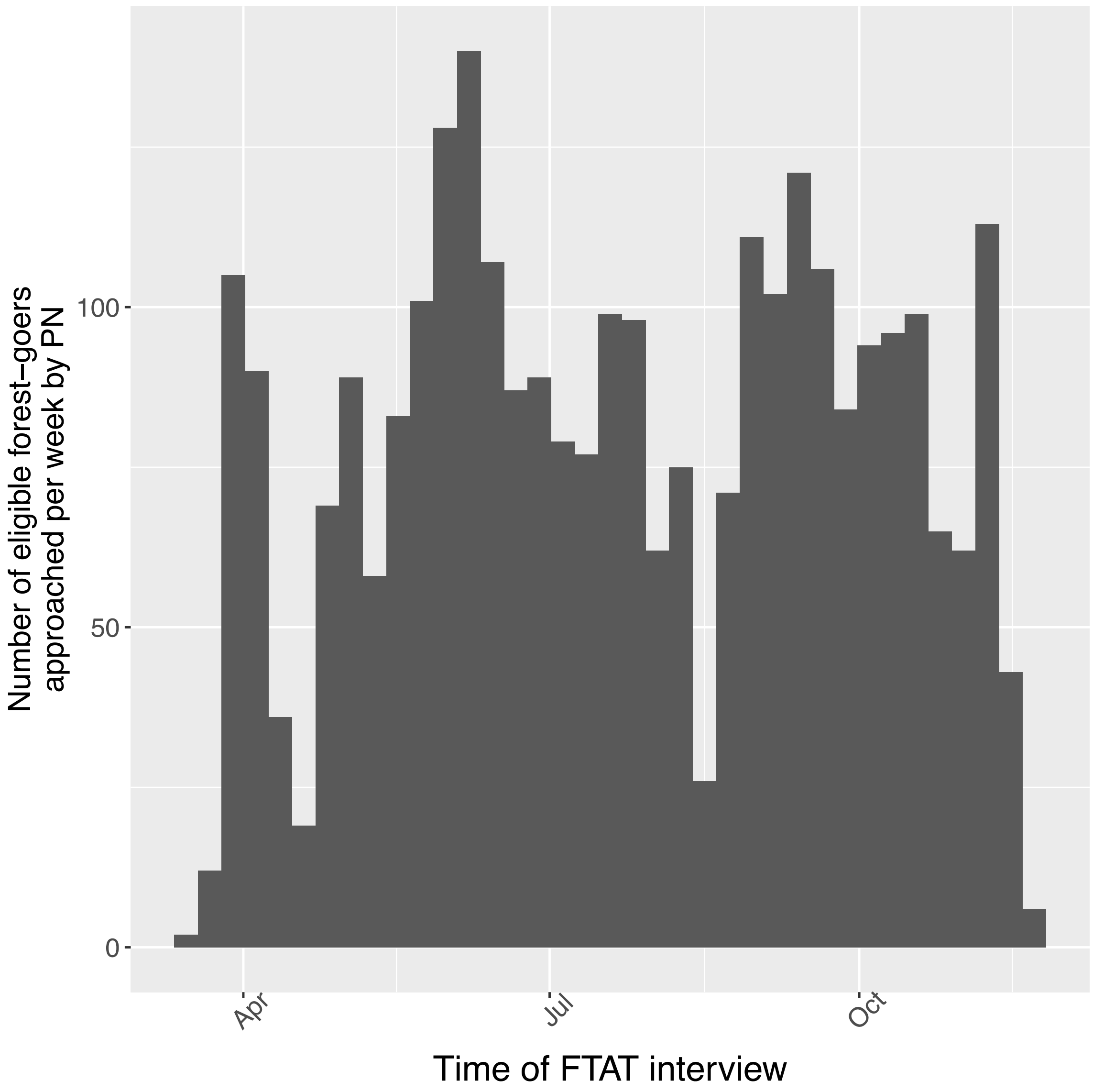

Figure S4.2 – FTAT HRP enrollment: *Enrollment of HRP individuals in FTAT survey over time.*

Supplementary Materials 5: Sensitivity analyses for population-based household survey PSE

In a first sensitivity analysis, we considered how the differences among criteria may lead to an underestimate of the PSE for the MTAT survey. Indeed, criterion A in the baseline and endline surveys maps onto criterion D in the MTAT survey, but the differences between criteria C and F and the absence of an equivalent for criteria B in MTAT may result in an undercount of HRP individuals in MTAT. Therefore, we calculated the proportion of HRP individuals missed in the baseline and endline surveys if only criteria A was to be used, and upweighted the count of HRP individuals in MTAT accordingly.

In a second sensitivity analysis, we attempted to adjust for potential selection bias because of absent households during surveys. Indeed, if absence was related to forest or agriculture activities, as was informally reported by survey teams, our PSE estimates would be biased, presumably downwards. In this sensitivity analysis, we replaced absent households by households with 25%, 50% or 75% household members qualifying as HRP. Absent and surveyed households were taken to be of the same size. This rather conservative sensitivity analysis estimates upper bounds for the PSE in each of the three population-based surveys and quantifies the possible impact of such selection bias. Adjustment for differences in HRP eligibility criteria across surveys, as discussed in the first sensitivity analysis, was also included here.

Table S5.1 presents the results of our sensitivity analyses. In the first sensitivity analysis, we estimated that 30% of HRP individuals would be missed if only criterion A was to be used in the baseline and endline surveys. As a result, we upweighted the MTAT HRP count (if only criteria D was to be used) to estimate 11.1% [8.6; 13.9] for the population proportion of HRP in MTAT. This is slightly higher than the 9.3% [7.2; 11.3] reported in the primary analysis (Table 3) but not massively different. In the second sensitivity analysis, we estimated an upper bound for each of the three population-based PSEs when attempting to adjust for selection bias and replaced absent households by households with 25%, 50% or 75% household members qualifying as HRP. At baseline, MTAT and endline respectively, 6.2%, 16.3% and 7.4% of surveyed households were absent.

	Primary analysis	Sensitivity analysis 1	Sensitivity analysis 2		
		Address differences in HRP's eligibility criteria among surveys	Absent households during surveys replaced by households where X% of households members are HRP individuals		
			25%	50%	75%
	% HRP [95% CI]	% HRP [95% CI]	% HRP [95% CI]	% HRP [95% CI]	% HRP [95% CI]
Baseline	16.2 [14.7; 17.7]	16.2 [14.7; 17.7]	16.7 [15.3; 18.2]	18.3 [16.9; 19.7]	19.8 [18.4; 21.3]
MTAT	9.3 [7.2; 11.3]	11.2 [9.3; 13.0]	13.4 [11.6; 15.0]	17.5 [15.9; 19.0]	21.6 [20.0; 23.1]
Endline	5.3 [4.4; 6.1]	5.3 [4.4; 6.1]	6.8 [5.9; 7.5]	8.6 [7.8; 9.3]	10.5 [9.6; 11.2]

Table S5.1 - *Results for the population-based survey method for population size estimation of HRP individuals. Sensitivity Analyses.*

Supplementary Materials 6: Sensitivity analyses for capture-recapture PSE

Three sensitivity analyses were conducted to strengthen the robustness of our results. The first two sensitivity analyses estimate a lower bound for our PSE by either relaxing the matching criteria or augmenting the eligibility criteria in FTAT. In the first one, matches with plus or minus 3 years for age and plus or minus 2 ordered education categories apart were additionally accepted in our algorithm to relax our matching constraints. In the second one, the eligibility criteria for FTAT were augmented to only include individuals who, when asked specifically which months of the year they tended to spend a night outside in the forest or in the rice field, listed one of the two months prior their FTAT interview (May and June for someone interviewed in June for instance).

The third sensitivity analysis attempts to assess and correct for potential matching errors among HRP individuals, either because of which identifying variables were selected or how they were used in the matching algorithm. Unlike conventional capture-recapture studies, some of our traps, namely the three population-based surveys, not only capture HRP but also non-HRP individuals. These can be considered captures of individuals living in the study area, regardless of their HRP status. Using these captures, the total population in the study area was estimated by running the same capture-recapture methodology with the same matching algorithm and the same identifying variables on the complete four survey lists. This estimate was compared to the actual total population, known from the census count, to infer how biased the capture-recapture PSE may be and correct it accordingly. Such correction relies on the reasonable assumption that matching errors between two individuals are as likely to happen in the general population as among HRP individuals.

Results from our first two sensitivity analyses, which can be viewed as tentative to estimate a lower bound, yielded PSE = 15,305 [13,886; 16,959] when matches with plus or minus 3 years of age and plus or minus 2 ordered education categories apart were allowed and PSE = 16,380 [14,555; 18,566] when FTAT eligibility criteria was augmented. In the third sensitivity analysis, trying to adjust for potential mismatch among HRP individuals because of which identifying variables were selected and how they were used in the matching algorithm, the M_t log-linear model estimated a total population in the study area of 52,739 [51,655; 53,878] which is close to the true 47,575 in the household census count. This discrepancy means that our matching algorithm slightly underestimates the true overlap among the four lists of HRP individuals. Assuming the degree of mismatching error is the same among the general population as among HRP individuals, correcting the estimate by a 1.11 factor would yield a PSE = 16,622 [14,911; 18,646]. All of these three sensitivity analyses yielded fairly similar PSEs and strengthened the robustness of our estimate.

Supplementary Materials 7: Details on record matching for population-based PSEs

First, each survey sample was restricted to participants who met the same HRP criteria applied in the population-based method. Four different lists of HRP individuals were therefore extracted: baseline, MTAT, endline and FTAT.

HRP individuals needed to be uniquely identified in a consistent manner across surveys in order to ascertain overlap. Age, sex, highest level of education, first name initial and home village were extracted as variables collected in all surveys with the potential to uniquely identify HRP individuals across surveys. Age was either self-reported or computed from date of birth and was rounded to years. Because surveys were conducted at different times, age was standardized across surveys using July 1st, 2018 (middle of MTAT) as reference. Education was self-reported using 6 categories (none, some primary school, completed primary school, some secondary school, completed secondary school, more than secondary school). Ethnicity was not used because it was unavailable for participants other than heads of household in the 3 cross-sectional surveys. Only the initials of the first and last names were reported in the FTAT survey while the complete first name was reported in the 3 cross-sectional surveys. Thus, names could only be matched based on the first initial: the initial was extracted from the complete first name after removing titles (e.g., “Miss”, “Mister”), which were identified in collaboration with a Lao consultant. Overall, the combination of these five identifying variables was unique for 99.5% of HRP individuals in all data sources (100% in baseline and endline, 99.5% in MTAT and 98.9% in FTAT).

HRP individuals from the 4 different lists were matched based on age, sex, level of education, first initial and home village. Matches with plus or minus 2 years for age and plus or minus 1 ordered education categories apart (or missing) were accepted. This flexibility was allowed because rounding age may have introduced errors and self-reported education was considered to be less reliable and prone to social desirability and recall biases with individuals potentially mixing up secondary and primary school or reporting school completion instead of some school in two different surveys. For names, records from cross-sectional surveys were matched with FTAT participants when the first initial extracted from the cross-sectional survey matched one of the initials reported in FTAT. Indeed, the first initial could not be isolated from the last initial in the FTAT survey. When a record appeared to match multiple records from another data source, perfect matches were favored. In 19% of all matches, ties persisted, and we randomly selected one of them.

Supplementary Materials 8: Methodological details on capture-recapture

Theory

The capture-recapture methodology, originating in wildlife ecology, relies on the overlap of animals captured on different trap occasions to estimate the unknown total population size. Animals captured in the first sample are marked and then released back in the population. In the second sample, there will be animals tagged, meaning they were captured in the first sample, and untagged animals. This results in a two-source capture-recapture dataset with two samples of respective size n_1 and n_2 comprising m_2 recaptures. The Petersen estimator[46] equates the proportion of tagged animals in the population and in the second sample to estimate the total population size of animals $\widehat{N} = n_1 \times n_2 / m_2$. If more than two samples are collected, the process is repeated, uniquely tagging captured individuals on each occasion before releasing them. The capture histories for each individual - e.g., 01011 for an animal captured on occasions 2, 4 and 5 but not on occasions 1 and 3 - are then analyzed to estimate the total population size.

Typically, the simple estimators rely on the key assumptions that 1) the population is closed to additions and deletions, 2) all animals are equally likely to be caught on each capture occasion and 3) marks are reliable to assess the capture histories. That said, advanced methods such as log-linear models[35–39], can relax some of those assumptions.

Here, the different surveys serve as traps and HRP individuals are "captured" when they participate in a survey. Hence, each of the four surveys represent a different capture occasion. Unique identifying variables can be used to track in which surveys individuals were captured.

Statistical analysis

The overlap among the 4 lists of HRP individuals was analyzed using log-linear models[35–39] available in the Rcapture[40] R package. Ubiquitous in the capture-recapture methodology, log-linear models leverage the overlap among lists to estimate the capture probability *p*, i.e the probability of being captured in a survey. In the simplest model M_0, the population is assumed to be closed with no in or out immigration and *p* is the same for all individuals and all traps, i.e surveys here. These stringent assumptions can be relaxed to model an open population or to allow heterogeneity in *p*, with additional parameters to be estimated, depending on the data available and assumptions researchers are willing to make. Three main sources of heterogeneity are discussed in the literature and available for modeling in the Rcapture[40] package. First, models M_t allow temporal dependance where the capture probability p_t can change between capture occasions, i.e surveys here. Second, models M_h allow the capture probability p_h to vary between individuals of the population. Third, trap dependence can be modeled in M_b where individuals' capture probability p_b can depend on their previous capture history. Any combination of the 3 sources of heterogeneity can be modeled such as in M_{th} where temporal dependence and individual heterogeneity are allowed but not trap dependence. Last, interaction terms can be included in log-linear models[47] when capture probabilities are correlated between two lists at a

population level - referred as list dependency in the epidemiological terminology[37]. The fit to the data, as indicated by the AIC or BIC, should guide model selection.

In our study, trap dependence does not make sense as the probability of being in one survey should not depend on whether or not an individual was in a previous survey. In particular, surveys' samples were randomly selected independently from one another. Heterogeneity in gender, age or ethnicity may definitely result in capture probability heterogeneity among HRP individuals. Our formative work indicated that spending a night outside in the forest or in the rice field was not stigmatized in the region, but some HRP criteria were answered by the head of the household, who may not be fully aware of the forest going habits of their household members. In addition, our capture instruments, i.e surveys, may not cover the whole spectrum of HRP forest-going activities equally and may therefore introduce some heterogeneity in the capture probabilities. For instance, HRP individuals traveling frequently in and out of the forest or through major forest entry points may be more likely to be ascertained in the FTAT survey. Surveys' questions also rely on a local understanding of what "sleeping", "forest", "home" or "sleeping outside in the forest away from home" mean and may result in different capture probabilities among HRP individuals.

The number of HRP individuals captured is expected to fluctuate across different months because of the influence of the dry and rainy seasons on livelihoods and forest or agricultural activities. In our modeling framework, we can conceptualize this temporal dependence in two ways. First, we considered a closed population where HRP individuals remain so all year long but where the probability of being captured and identified as a HRP in a survey varies across surveys because of varying probability of spending a night outside in a given month (M_t models). Second, we considered an open population with HRP individuals migrating in and out of the population depending on whether or not they spent a night outside in a given month. Both models estimated the same PSE, i.e the total population size of the forest-going HRP in the study area during the study period, which covers 1 full year between December 2017 and November 2018. As noted by Pollock[48], one of the founders of the capture-recapture methodology, the distinction between open and closed population may be artificial and mainly resides in their aptitude to estimate different parameters. Closed population models focus on estimating capture probabilities whereas open population models focus on estimating migration rates.

Baillargeon and Rivest[40] provide a nice illustration of the log-linear models fitting process with the simplest model for a closed population M_0. For a dataset with t capture occasions, 2^t -1 capture histories, ω, are observables. Again, with t =5, 01011 is the capture history for an individual captured on occasions 2, 4 and 5 but not on occasions 1 and 3. In M_0, which has a single capture probability p, the probability for an individual to experience a capture history ω is

$$P(\omega) = p^{\sum \omega_j}(1-p)^{t-\sum \omega_j}$$

where $\sum \omega_j$ is the number of times the individual is captured. Therefore, the expected number of units in the population with capture history ω is given by:

$$\begin{aligned}\mu_\omega &= N \times P(\omega) \\ &= Np^{\sum \omega_j}(1-p)^{t-\sum \omega_j} \\ &= \exp\left(\log\left(N(1-p)^t\right) + \sum \omega_j \log\left(\frac{p}{1-p}\right)\right)\end{aligned}$$

The log-linear model therefore fits E[Y] = exp(α + Xβ), where Y is the vector of observed capture history frequencies and X is a vector defined by $\sum \omega_j$. Then, the total population size is estimated as $\widehat{N} = n + \exp(\hat{\alpha})$ where n is the total number of units captured in the data. This is because:

$$\exp(\alpha) = \exp(log(N(1-p)^t)) = N(1-p)^t = N \times P(\omega_0) = \mu_0$$

where ω_0 is the unobservable capture history of zero captures and μ_0 is the expected number of units never captured. Estimation is done using maximum likelihood for a Poisson count random variable for the number of individuals with a certain capture history. See Rivest and Baillargeon[47] for more details on other log-linear models.

Finally, Table S8.1 shows how the expected number of individuals with a certain capture history is parametrized in log-linear models for closed and open population models[36]. For simplicity, three (instead of four in our context) source models are shown.

	Expected number of individuals with certain capture histories for various models		
Capture history	Closed population (M0)	Closed population with temporal dependence (Mt)	Open population
111	$N \times p \times p \times p$	$N \times p_1 \times p_2 \times p_3$	$N \times p_1 \times \phi_1 p_2 \times (1-\chi_2)$
011	$N \times (1-p) \times p \times p$	$N \times (1-p_1) \times p_2 \times p_3$	$N \times (1-p_1) \times \phi_1 \psi_1 p_2 \times (1-\chi_2)$
101	$N \times p \times (1-p) \times p$	$N \times p_1 \times (1-p_2) \times p_3$	$N \times p_1 \times \phi_1(1-p_2) \times (1-\chi_2)$
001	$N \times (1-p) \times (1-p) \times p$	$N \times (1-p_1) \times (1-p_2) \times p_3$	$N \times (1-p_1) \times \phi_1 \psi_1 (1-p_2) \times \psi_2(1-\chi_2)$
110	$N \times p \times p \times (1-p)$	$N \times p_1 \times p_2 \times (1-p_3)$	$N \times p_1 \times \phi_1 p_2 \times \chi_2$
010	$N \times (1-p) \times p \times (1-p)$	$N \times (1-p_1) \times p_2 \times (1-p_3)$	$N \times (1-p_1) \times \phi_1 \psi_1 p_2 \times \chi_2$
100	$N \times p \times (1-p) \times (1-p)$	$N \times p_1 \times (1-p_2) \times (1-p_3)$	$N \times p_1 \times \lambda_1 \phi_1 * (1-p_2) \times \chi_2$

Table S8.1 - *Parametrization for the expected number of individuals with certain capture histories for various three source capture-recapture models. The probability of being captured in a survey is allowed to vary across the 3 surveys as p1, p2 and p3 but is constant in* M_0 *model. Other parameters include* ϕ_i*, the probability that an individual survives from the* i^{th} *to* $(i+1)^{th}$ *sample;* χ_i*, the probability that an individual is not seen after the* i^{th} *sample;* $1/\psi_i$*, the probability that an individual alive and unmarked in the population at the time of the* $(i+1)^{th}$ *sample was in the population at the time of the* i^{th} *sample; and* $1/\lambda_i$*, the probability that an individual alive in the population at the time of the* i^{th} *sample but not observed thereafter, is still alive in the population at the time of the* $(i+1)^{th}$*. Note the substitution* $\lambda_i = \chi_i/(\phi_i(1-p_{i+1})\chi_{i+1})$.

Supplementary Materials 9: Diagnostic test for heterogeneity in log-linear models

Figure S9.1 shows a diagnostic test confirming heterogeneity between individuals in terms of their capture probability does not need to be included in the model. Work from Lindsey (1986) and Rivest (2007) shows that this plot for f_i, the number of units captured on i different occasions, should be concave upward in the presence of heterogeneity. On the other hand, linearity indicates heterogeneity does not need to be accounted for in the model.

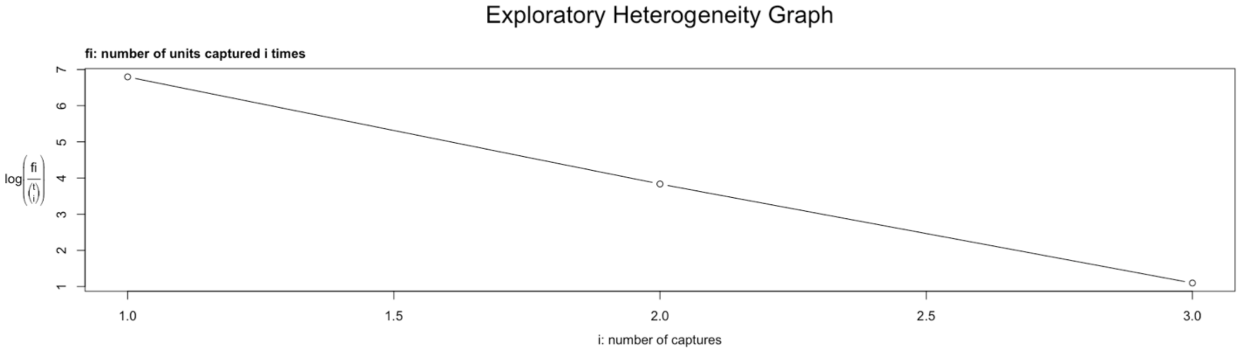

Figure S9.1 - *Diagnostic test for heterogeneity: linearity indicates that individual heterogeneity in terms of their capture probability is not needed in the models.*

Supplementary Materials 10: subdividing the PSE by type of risk behavior/activity

Based on individuals' responses to the criteria in Table 1, we split the definition of HRP based upon forest-related activities or agriculture-related activities. For instance, in this secondary analysis, criterion A was split between the following 2 sub-criteria:

- A_Agriculture: During the past month, stayed overnight away from home AND reason for the absence was working in the *rice field or plantation* in this province or another province
- A_Forest: During the past month, stayed overnight away from home AND reason for the absence was working in the *forest* in this province or another province

Criterion C could not be split between forest and agriculture activities because of how the question was framed. In a sensitivity analysis, individuals meeting criterion C were all allocated to either the forest sub-category or the agriculture sub-category, producing liberal and conservative estimates for the population size of HRP in the two sub-categories. Also note that criterion F only pertains to HRP individuals being identified because of their forest activities. Last, the FTAT HRP criteria could not be split either because of the eligibility criteria in the FTAT intervention.

Table S10.1 and S10.2 show the results from the household survey methods when splitting our definition of HRP between those that were identified as HRP because of forest-related activities or because of agriculture-related activities. Results from the sensitivity analysis are also reported.

	Estimate	% HRP [95% CI]	PSE [95% CI]
Baseline	HRP Agriculture (Conservative)	7.2 [5.9; 8.4]	2,164 [1,794; 2,539]
Baseline	HRP Agriculture (Liberal)	15.6 [14.7; 17.1]	4,702 [4,246; 5,156]
MTAT	HRP Agriculture	6.6 [4.8; 8.5]	2,006 [1,436; 2,568]
Endline	HRP Agriculture (Conservative)	3.8 [3.0; 4.6]	1,147 [910; 1,385]
Endline	HRP Agriculture (Liberal)	5.2 [4.3; 6.1]	1,569 [1,308; 1,821]

Table S10.1 - *Results for the population-based survey method for population size estimation of agriculture-related HRP individuals*

	Estimate	% HRP [95% CI]	PSE [95% CI]
Baseline	HRP Forest (Conservative)	4.9 [4.1; 5.8]	1,496 [1,238; 1,756]
Baseline	HRP Forest (Liberal)	13.8 [12.4; 15.2]	4,168 [3,743; 4,597]
MTAT	HRP Forest	9.0 [7.0; 11.0]	2,727 [2,124; 3,338]
Endline	HRP Forest (Conservative)	0.3 [0.2; 0.5]	100 [50; 152]
Endline	HRP Forest (Liberal)	4.3 [3.5; 5.1]	1,302 [1,058; 1,550]

Table S10.2 - *Results for the population-based survey method for population size estimation of forest-related HRP individuals*

In conclusion, the wording of surveys questions did not allow for a clear distinction in HRP criteria and the range of estimates was too wide to be informative.
